# Supplementary material for: Diatomic iron nanozyme with lipoxidase-like activity for efficient inactivation of enveloped virus
Source: Nat Commun. 2023 Nov 11;14:7312. doi: 10.1038/s41467-023-43176-4 (PMC10640610; doi:10.1038/s41467-023-43176-4)
Supplement: Supplementary file 1 — Supplementary Information [file 41467_2023_43176_MOESM1_ESM.pdf]

Supplementary Information

Diatomic Iron Nanozyme with Lipoxidase-like Activity for Efficient Inactivation of Enveloped Virus

Beibei Li[a,c,d,#], Ruonan Ma[b,#], Lei Chen[b,e,#], Caiyu Zhou[b,#], Yu-Xiao Zhang[a], Xiaonan Wang[b], Helai Huang[a], Qikun Hu[a], Xiaobo Zheng[c], Jiarui Yang[c], Mengjuan Shao[f], Pengfei Hao[g], Yanfen Wu[a], Yizhen Che[a], Chang Li[g], Tao Qin[f], Lizeng Gao[b,*], Zhiqiang Niu[a,*], and Yadong Li[c]

[a]State Key Laboratory of Chemical Engineering, Department of Chemical Engineering, Tsinghua University, Beijing 100084, China

[b]CAS Engineering Laboratory for Nanozyme, Institute of Biophysics, Chinese Academy of Sciences, Beijing, 100101, China

[c]Department of Chemistry, Tsinghua University, Beijing 100084, China.

[d]Henan Key Laboratory of Polyoxometalate Chemistry, College of Chemistry and Molecular Sciences, Henan University, Kaifeng, Henan 475004, China

[e]Department of Pharmacology, School of Medicine, Institute of Translational Medicine, Yangzhou University, Yangzhou 225001, China

[f]College of Veterinary Medicine, Yangzhou University, Yangzhou 225001, China

[g]Research Unit of Key Technologies for Prevention and Control of Virus Zoonoses, Chinese Academy of Medical Sciences, Changchun Veterinary Research Institute, Chinese Academy of Agricultural Sciences, Changchun 130000, China

[#]These authors contributed equally: Beibei Li, Ruonan Ma, Lei Chen, Caiyu Zhou

*e-mail: gaolizeng@ibp.ac.cn; niuzq@tsinghua.edu.cn

Table of Contents

1. Characterization
    a) Electron microscopy
    b) X-ray based structure characterization
    c) Single crystal analysis of Fe_2L complex
    d) EPR test
2. Supplementary Figures 1−16
3. Supplementary Tables 1−4
4. Supplementary References

1. Characterization

a) Electron microscopy

Cold field emission scanning electron microscope (SEM) images were collected on Hitachi SU-8000. Transmission electron microscopy (TEM) images were taken by Hitachi HT7700. The high-resolution HAADF-STEM characterization was performed using a probe aberration-corrected microscope (JEOL JEM-ARM200F) equipped with cold emitter operating at 300 kV. The attainable spatial resolution of this microscope is 78 pm.

b) X-ray based structure characterization

Powder X-ray diffraction (XRD) patterns were recorded on a Rigaku RU-200b X-ray powder diffractometer with Cu-Kα radiation (λ = 1.5418 Å). Metal content analysis was measured by a Thermo IRIS Intrepid II ICP-OES. Time of flight secondary ion mass spectrometry (TOF-SIMS) measurements were performed on TOF.SIMS 5-100 (ION-TOF GmbH) in positive ionization mode.

XPS measurements were carried out on an Omicron XPS System using Al Kα X-rays as the excitation source at a voltage of 15 kV and a power of 300 W. Binding energies were calibrated by setting the measured binding energy of C 1s to 284.8 eV.

The XAFS spectra at Fe *K*-edge were acquired at the 1W1B station in Beijing Synchrotron Radiation Facility (BSRF), operated at 2.5 GeV with a maximum current of 250 mA. The data for Fe_2 DAC and Fe SAC were recorded in fluorescence excitation mode using a Lytle detector. Iron foil, iron oxide, and Fe_2L complex were used as reference materials and measured in transmission mode using an ionization chamber. The acquired EXAFS data were processed according to the standard procedures using the ATHENA program integrated within the IFEFFIT (1.2.12) software packages.[1] The κ^2-weighted EXAFS spectra $\chi(k)$ were Fourier-transformed in a k-range of 3.0-12.5 $Å^{-1}$ for the metal *K*-edge with a Kaiser-Bessel window function.

c) Single-crystal analysis of Fe_2L complex

Single-crystal X-ray diffraction (SCXRD) data of Fe_2L complex was collected at 173 K on a SuperNova charge-coupled device (CCD) X-ray diffractometer, with Cu-Kα radiation (λ = 1.54184 Å). The crystal structure was resolved by directed methods and refined by full-matrix least squares fitting

on F^2 using the *SHELXL-2018* software package[2] and *OLEX2* program[3]. All non-hydrogen atoms were refined anisotropically. Hydrogen atoms were generated geometrically. Elemental analysis was made out on Vario EL III Elemental Analyzer (Elementar, Germany) equipped with a thermal conductivity detector (TCD).

d) EPR test

Electron paramagnetic resonance (EPR) was performed on ESR spectrometer (Bruker A300). 5,5-Dimethyl-l-pyrroline N-oxide (DMPO) was used as trapping agent.

2. Supplementary Figures 1−16

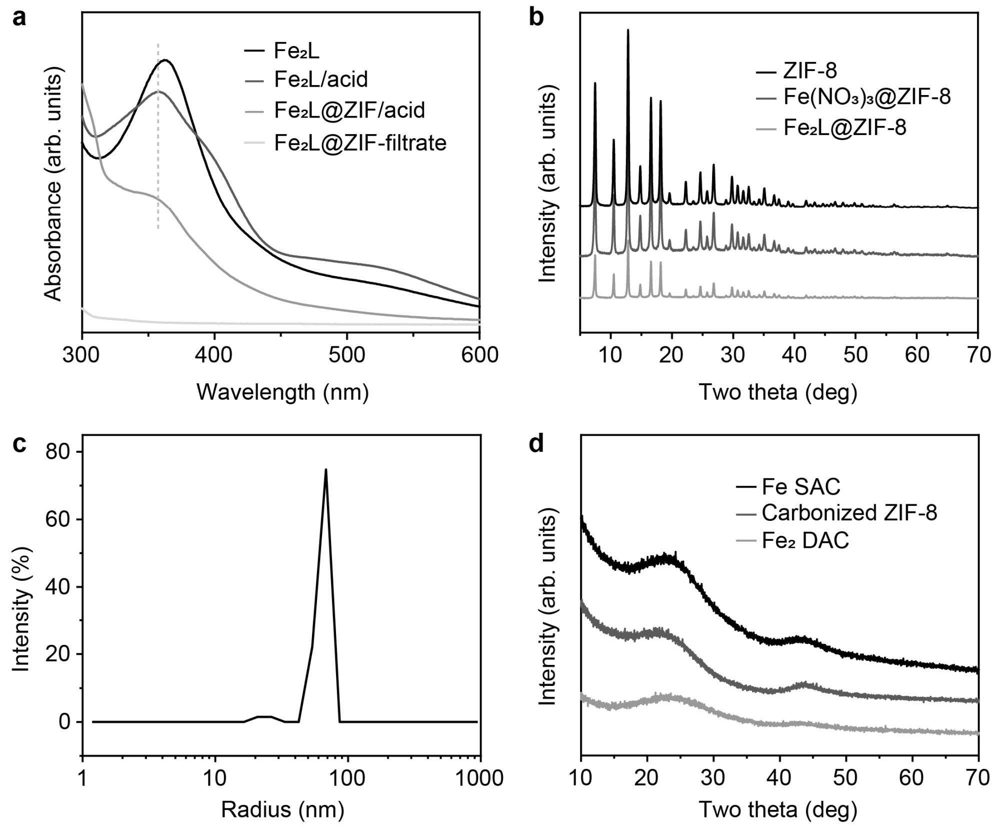

Supplementary Fig. 1. a, UV-Vis spectra of Fe_2L, Fe_2L after adding HAc, filtrate of Fe_2L@ZIF-8, and filtrate of Fe_2L@ZIF-8 after adding HAc. The results demonstrate that Fe_2L are encapsulated inside ZIF-8 (see more discussion in the Methods). **b,** Powder X-ray diffraction (PXRD) patterns of Fe_2L@ZIF-8, $Fe(NO_3)_3$@ZIF-8 and pure ZIF-8. **c,** The hydrodynamic diameter distribution of Fe_2 DAC nanozyme determined by dynamic light scattering (DLS). **d,** PXRD patterns of Fe_2 DAC, Fe SAC and carbonized ZIF-8.

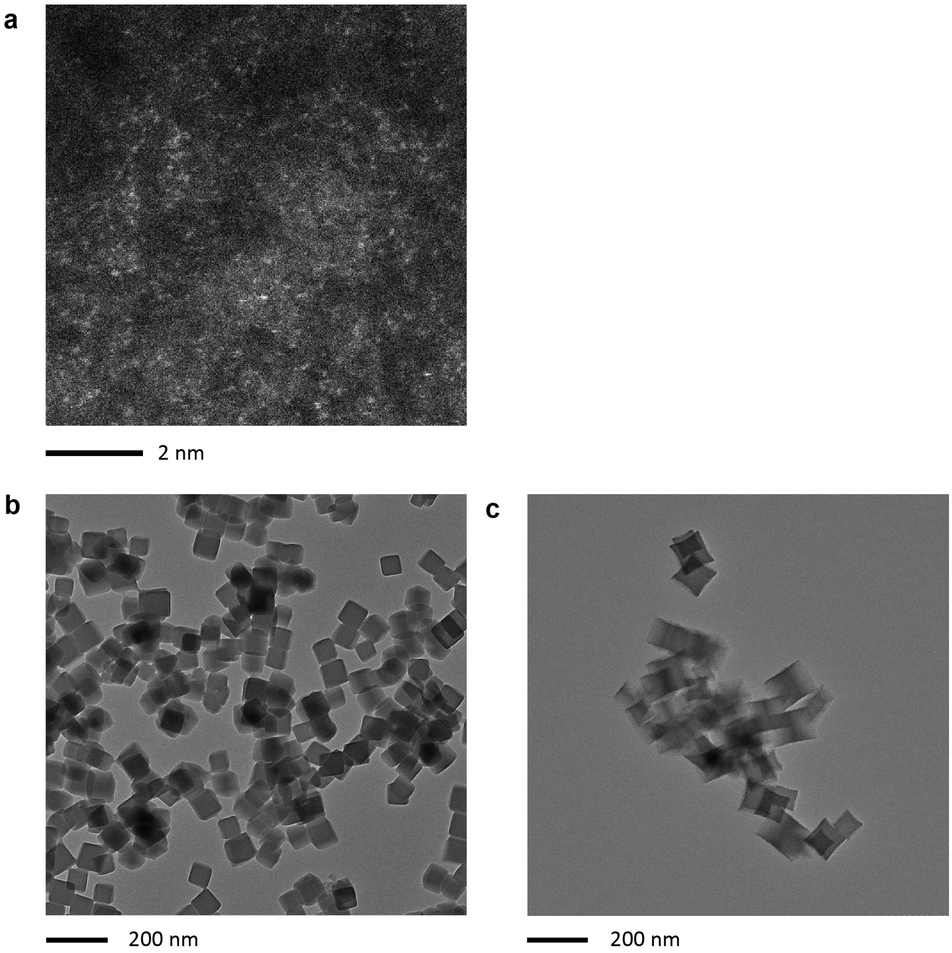

Supplementary Fig. 2. a, AC HAADF-STEM image of Fe SAC. **b-c,** Morphology of Fe_2L@ZIF-8 before (**b**) and after (**c**) pyrolysis. Three times each experiment was repeated independently with similar results, and representative images are presented.

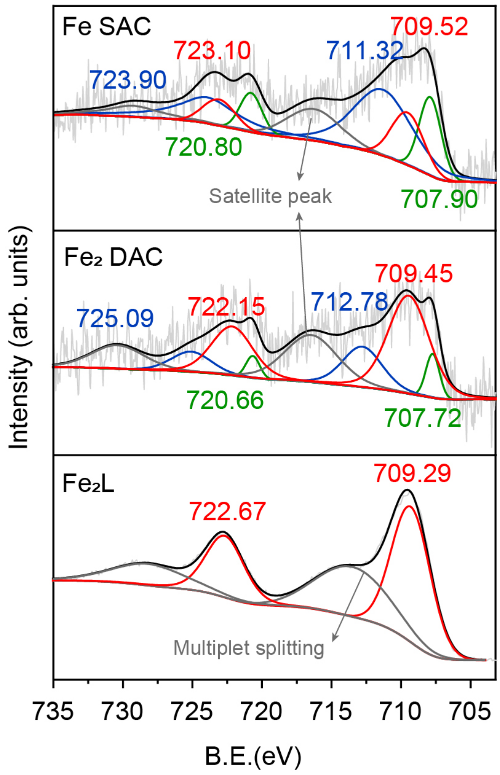

Supplementary Fig. 3. Fe 2*p* XPS spectra for Fe_2L, Fe_2 DAC and Fe SAC. [4-6]

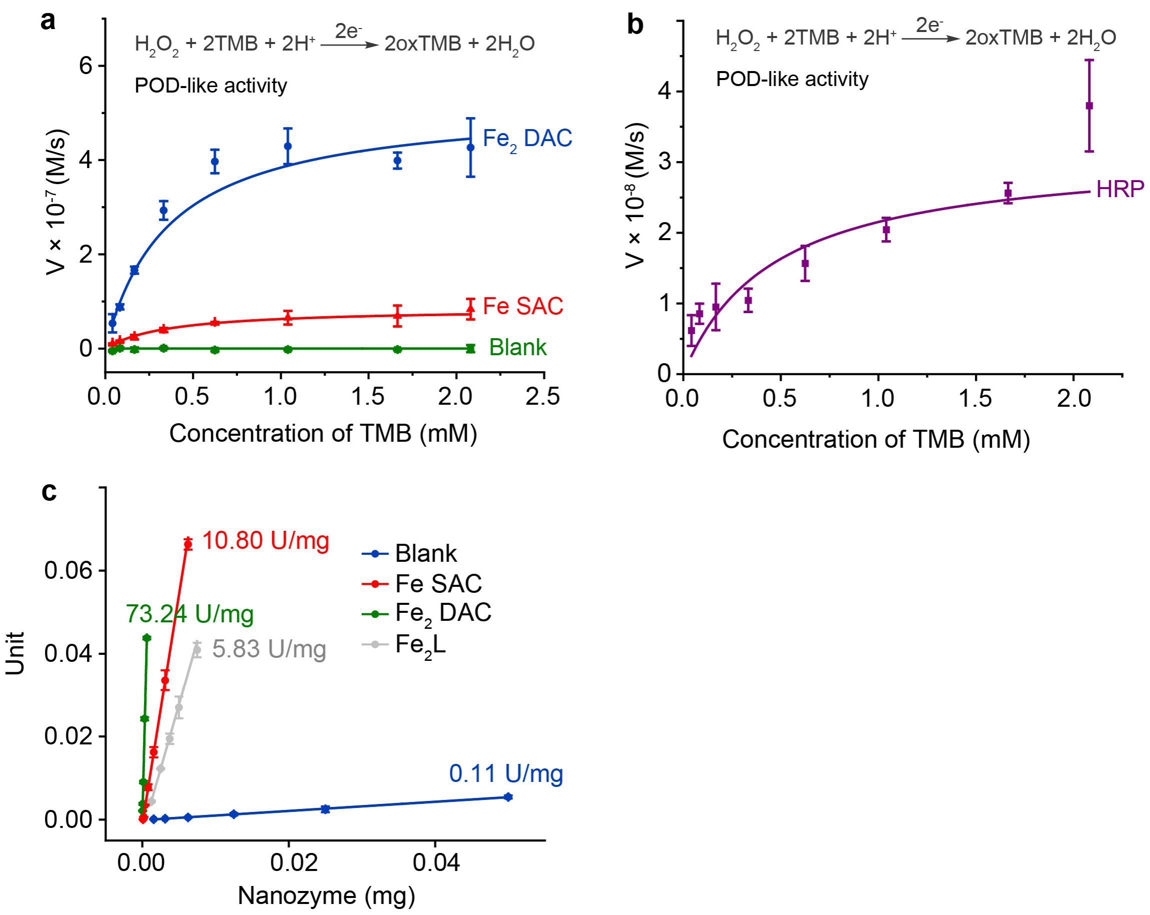

Supplementary Fig. 4. POD-like activity of Fe_2 DAC. a, Kinetics for POD-like activity of Fe_2 DAC, Fe SAC, and blank. **b,** Kinetics for POD-like activity of HRP. **c,** The specific POD-like activities (U mg^{-1}) of Fe_2 DAC, Fe SAC, Fe_2L, and blank (carbonized-ZIF-8). The nanozyme activity (U) is defined as the amount of nanozyme that converts 1 μmol of substrate per minute. The specific activities (U mg^{-1}) were determined by plotting the nanozyme activities against their weight and calculating the slopes of the data. The error bars represent standard deviations (SD) for three independent measurements.

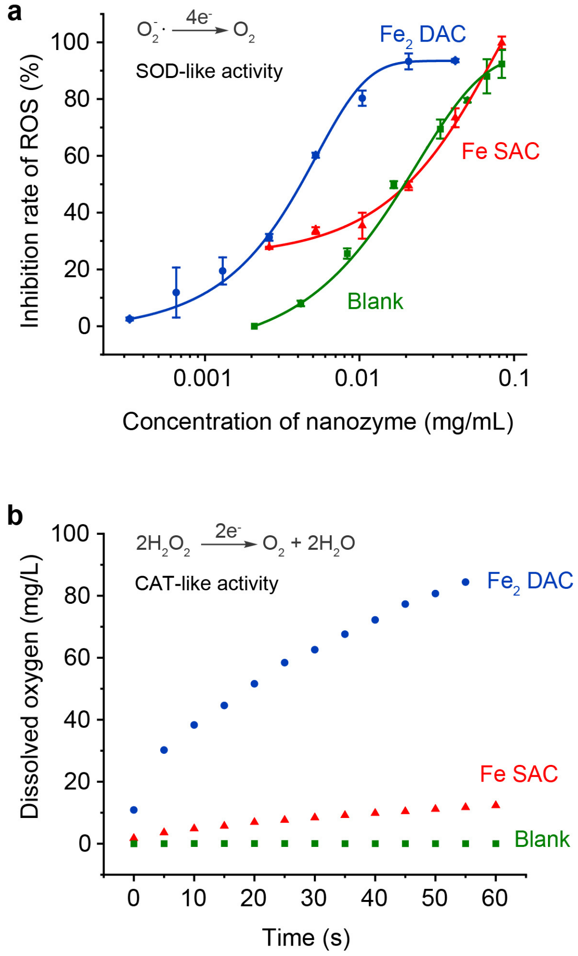

Supplementary Fig. 5. SOD-like and CAT-like activities of Fe_2 DAC. a, Kinetics for SOD-like activity of Fe_2 DAC, Fe SAC, and blank. **b,** Kinetics for CAT-like activity of Fe_2 DAC, Fe SAC, and blank. The error bars represent standard deviations for three independent measurements.

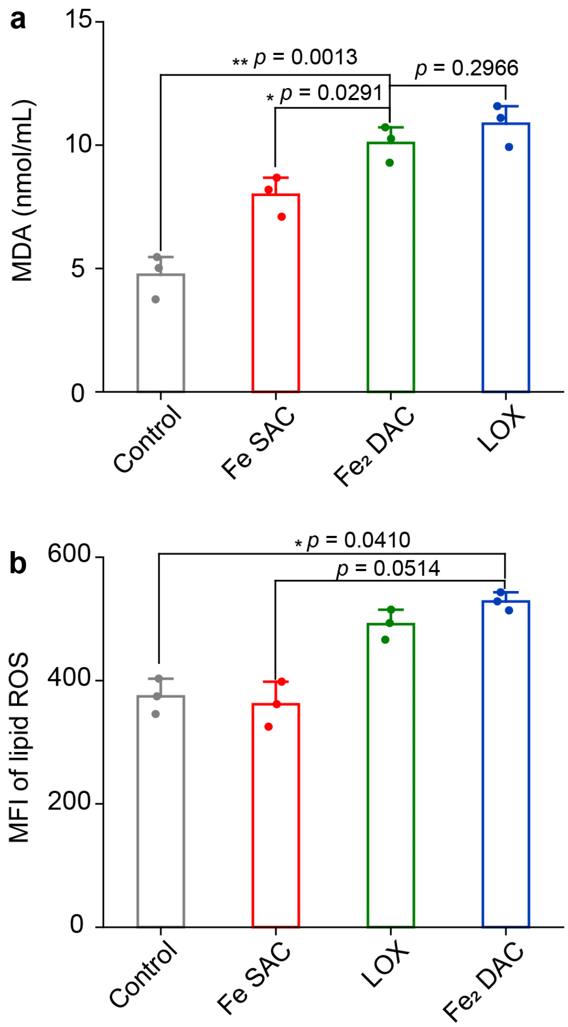

Supplementary Fig. 6. Detection of lipid peroxidation. Levels of **a,** MDA and **b,** lipid ROS (BODIPY 581/591 C11 probe) after liposomes treated by Fe_2 DAC, Fe SAC, and LOX. Data are presented as means±SD (n=3 independent measurements). The significant difference was evaluated by a two-tailed unpaired t-test. $*p < 0.05$ and $**p < 0.01$.

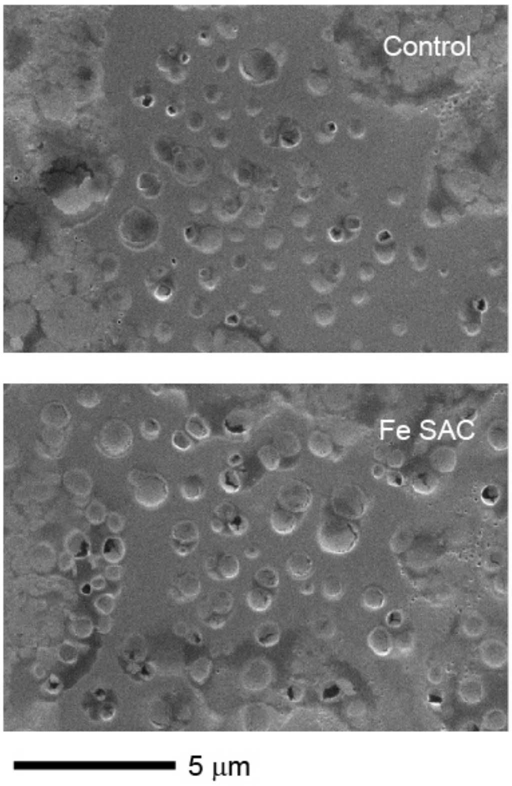

Supplementary Fig. 7. SEM images of liposomes treated by Fe SAC (500 μg/mL). Three times each experiment was repeated independently with similar results, and representative images are presented.

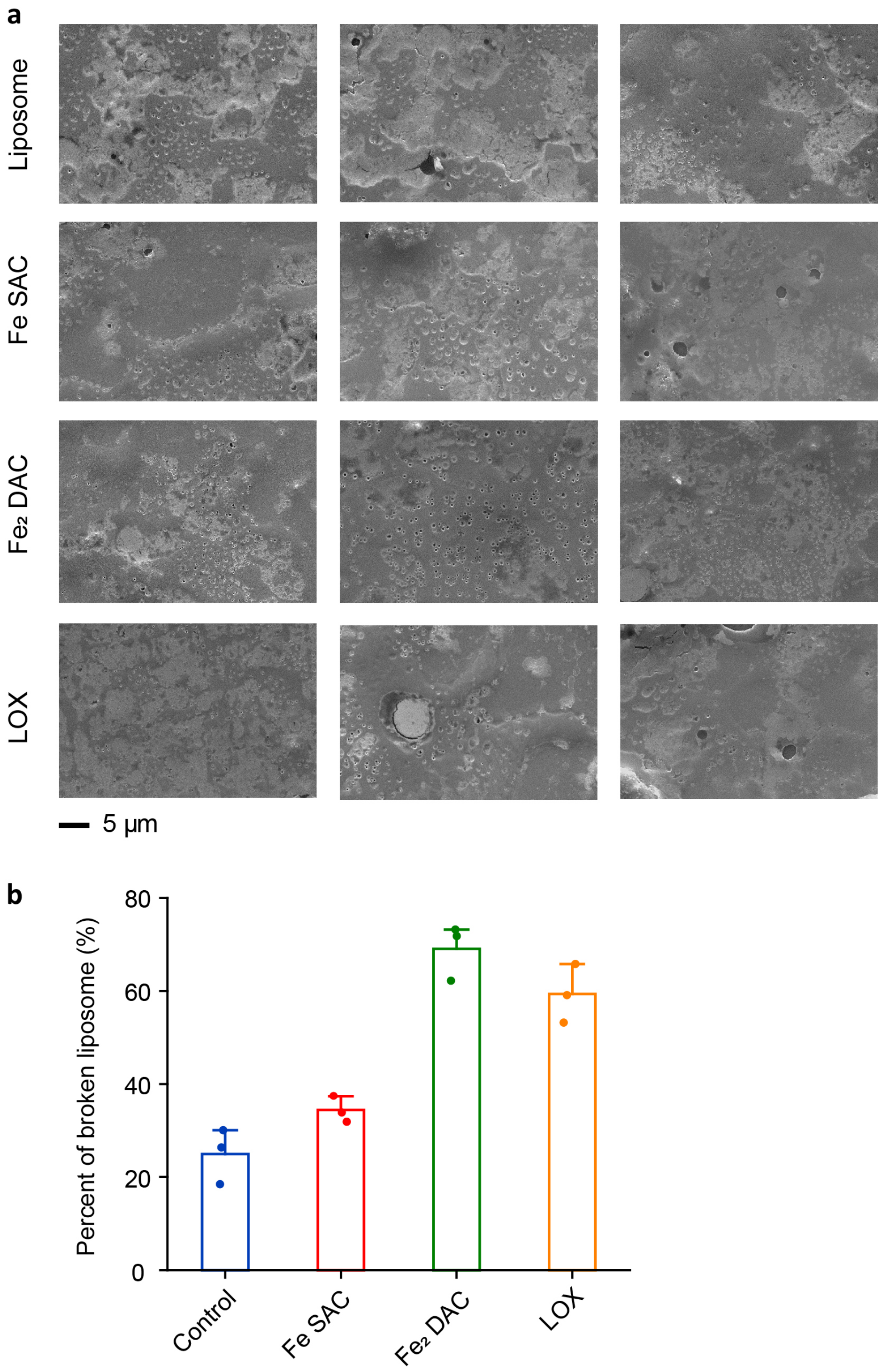

Supplementary Fig. 8. Destruction of liposomes. a, SEM images and **b,** quantitative statistics of liposomes treated by Fe_2 DAC, Fe SAC and LOX (n ⩾ 100). All the experiments have been repeated for three times independently and the data are presented as means±SD (n=3).

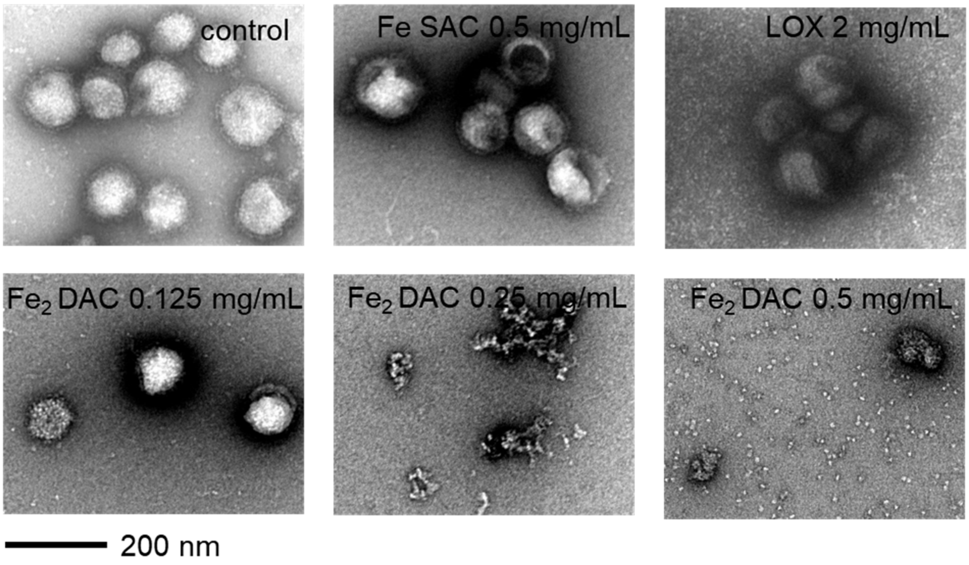

Supplementary Fig. 9. TEM images of H1N1 IAVs treated with Fe SAC (0.5 mg/mL), LOX (2 mg/mL) and variable concentrations of Fe_2 DAC. Three times each experiment was repeated independently with similar results, and representative images are presented.

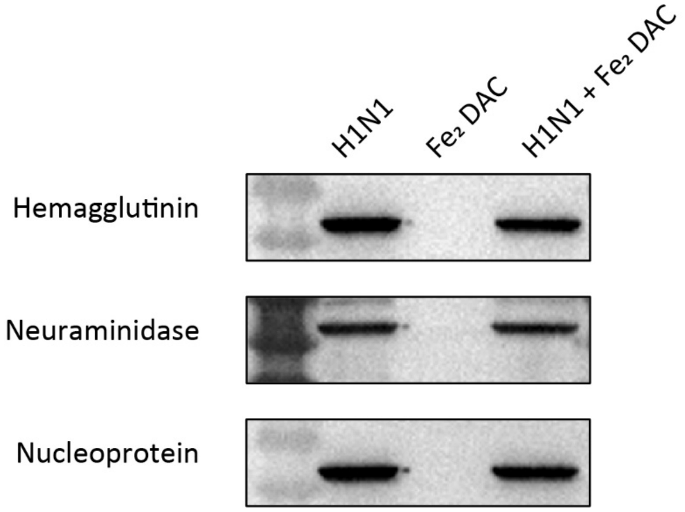

Supplementary Fig. 10. Western blot of hemagglutinin (HA), neuraminidase (NA), and nucleoprotein (NP) proteins of H1N1 IAVs, Fe_2 DAC, and Fe_2 DAC-treated H1N1 IAVs without incubation.

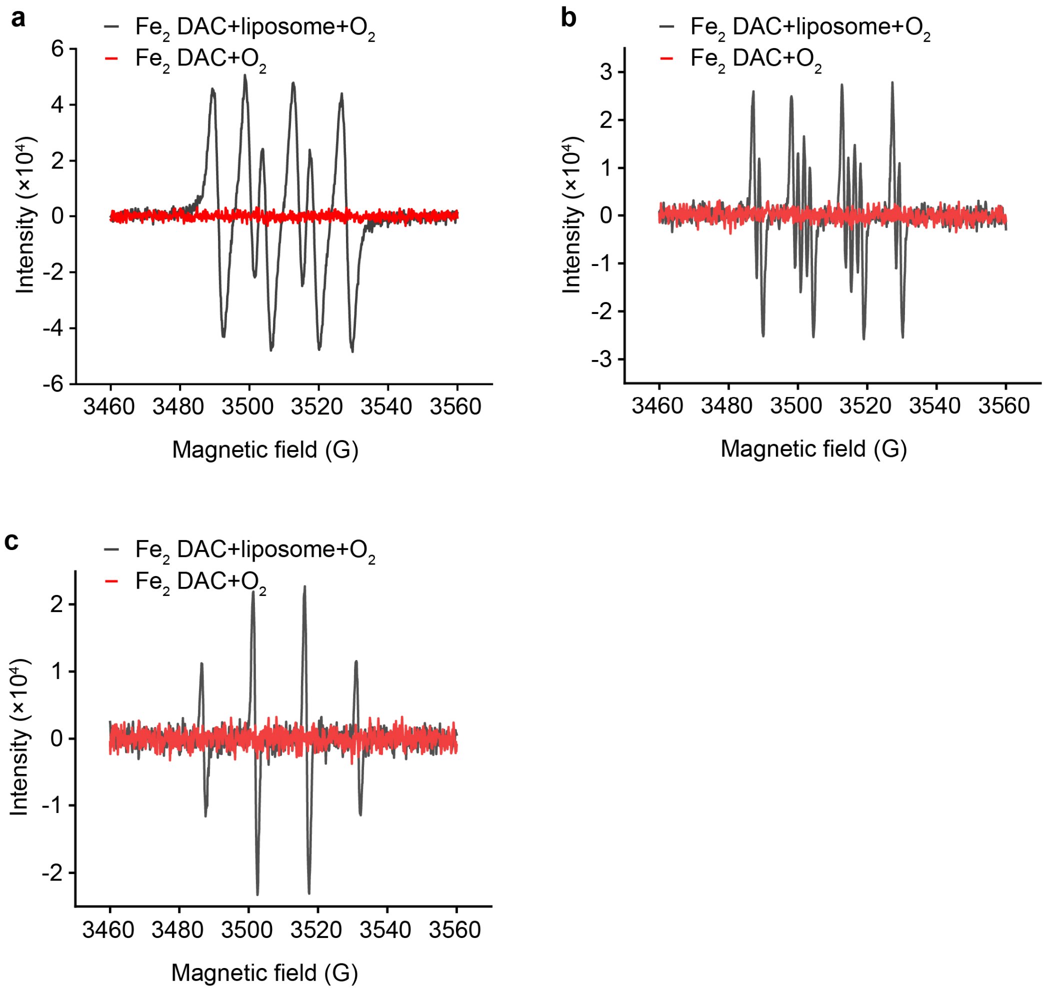

Supplementary Fig. 11. EPR analysis of Fe_2 DAC-treated liposome. The EPR signals of **a**, DMPO-•O_2^- adducts, **b**, DMPO-•O_2H adducts and **c**, DMPO-•OH adducts in the catalytic system of Fe_2 DAC-treated liposome and related controls under oxygen atmosphere.

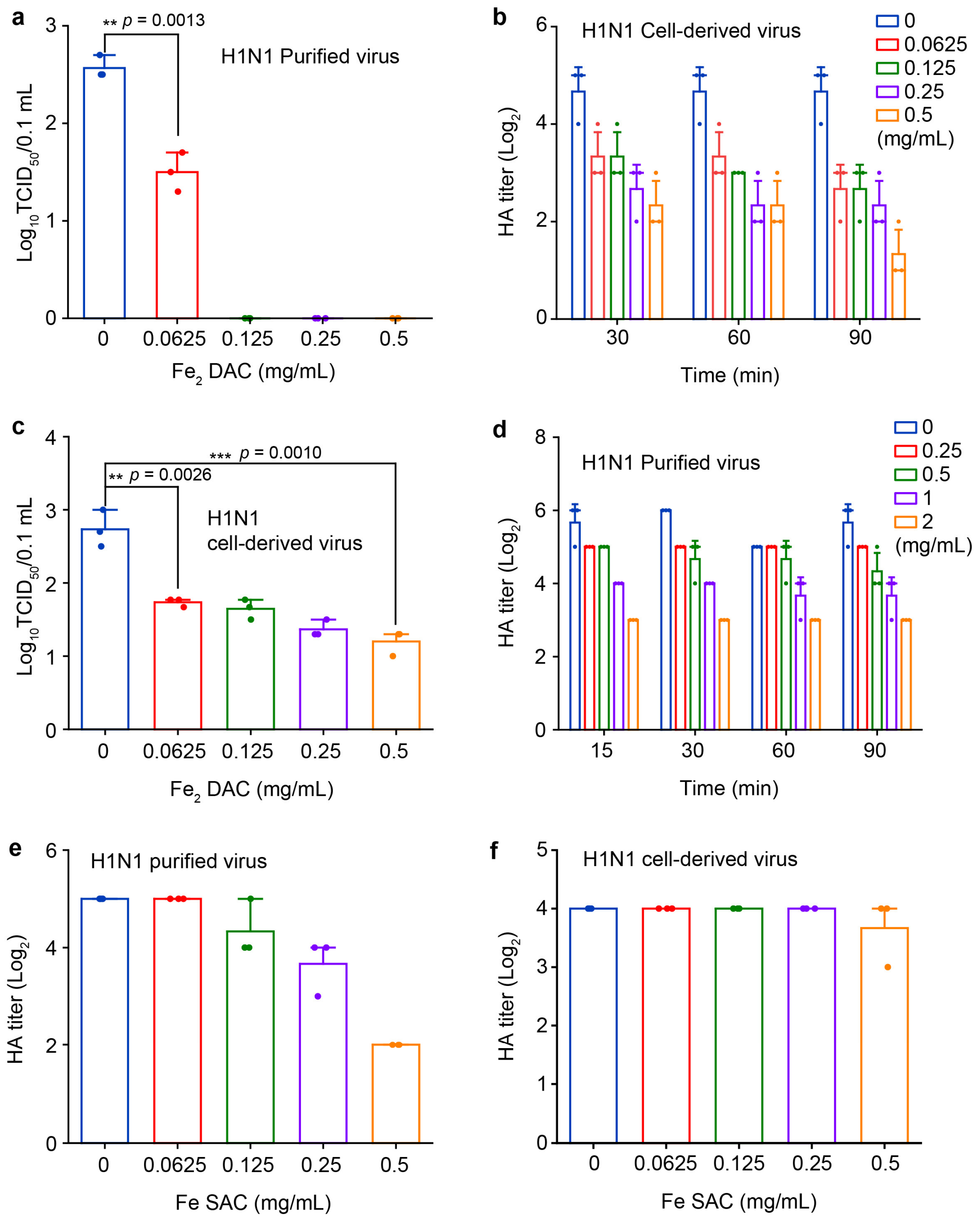

Supplementary Fig. 12. a, $TCID_{50}$ titer of Fe_2 DAC-treated H1N1 IAVs (purified virus) under 90 min. **b,** HA titer of Fe_2 DAC-treated H1N1 (cell-derived) IAVs, under 30/60/90 min. **c,** $TCID_{50}$ titer of Fe_2 DAC-treated H1N1 IAVs (cell-derived) under 90 min. **d,** HA titer of LOX-treated H1N1 (purified virus) IAVs, under 15/30/60/90 min. **e,** HA titer of Fe SAC-treated H1N1 (purified virus) IAVs under 90 min. **f,** HA titer of Fe SAC-treated H1N1 (cell-derived) IAVs under 90 min. All the data are presented as means±SD (n=3 independent measurements). The significant difference was evaluated by a two-tailed unpaired t-test. $^{**}p < 0.01$, $^{**}p < 0.01$ and $^{***}p < 0.001$.

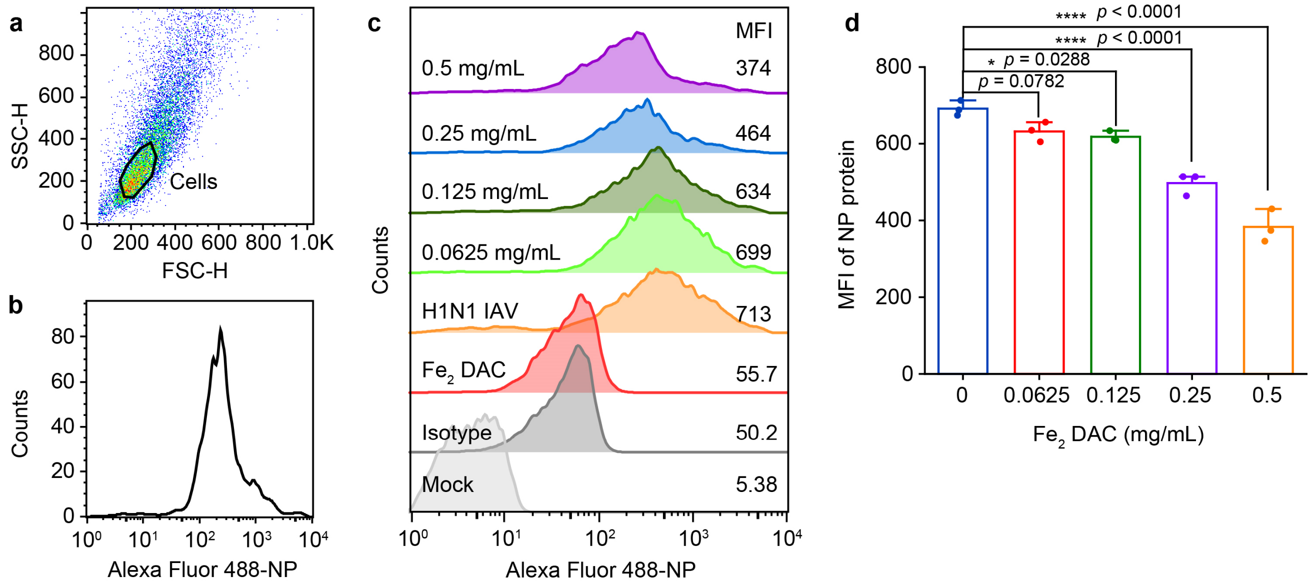

Supplementary Fig. 13. Detection of NP protein expression in M90 cells. a,b, Gating strategy used in flow cytometry analysis to detect NP+ M90 cells. Cell debris were excluded by SSC-H and FSC-H gating (Cells). Mean fluorescence intensity of NP protein on M90 cells was calculated by adding statistic Mean of Alexa Fluor 488, corresponding to (c) and (d). **c,** Flow cytometry of NP protein expression in M90 cells. **d,** Mean fluorescence intensity (MFI) of NP protein expression in (c). Data are presented as means±SD (n=3 independent measurements). The significant difference was evaluated by a two-tailed unpaired t-test. $*p < 0.05$ and $****p < 0.0001$.

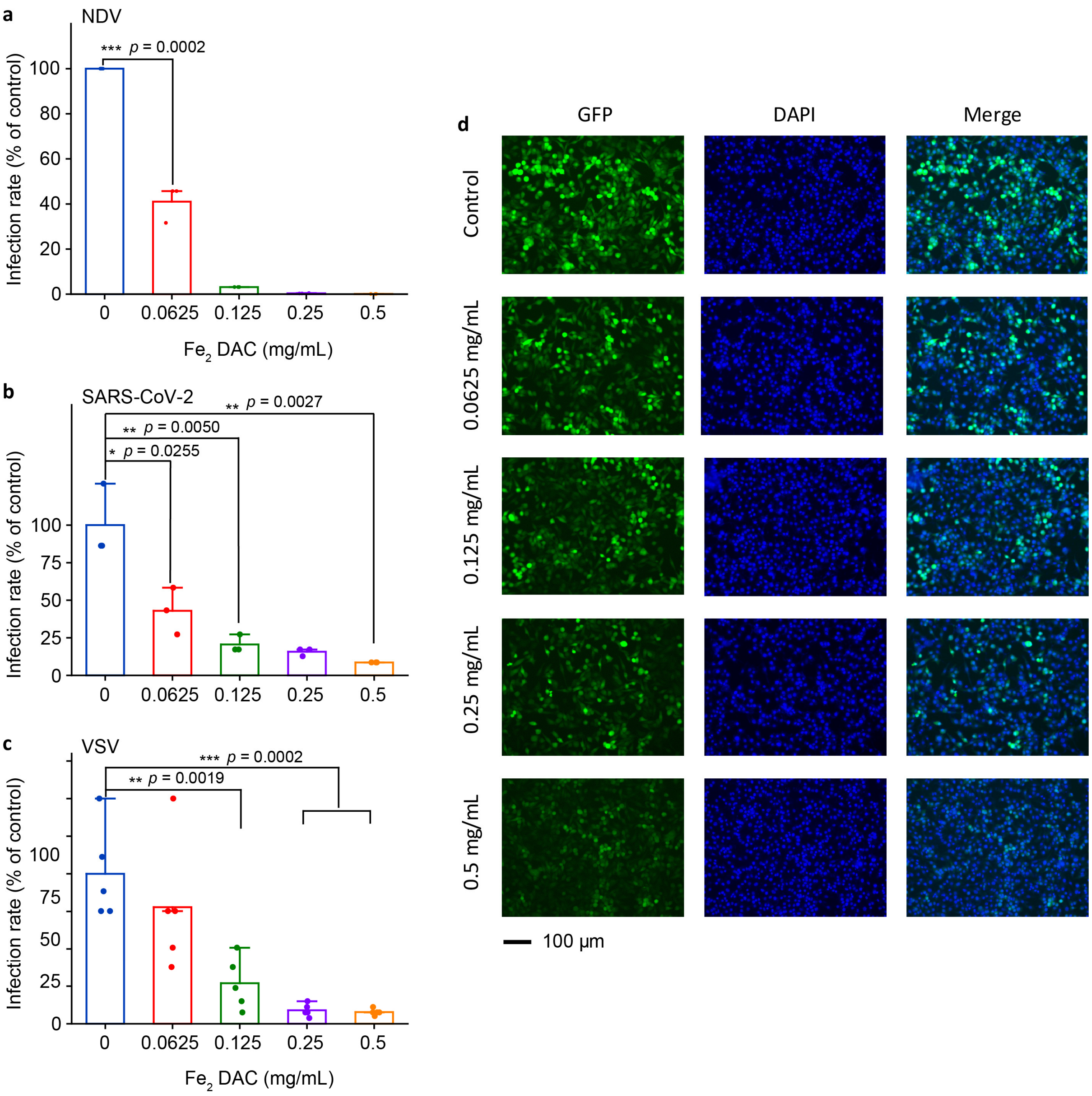

Supplementary Fig. 14. Antiviral effect of Fe_2 DAC to enveloped virus. Antiviral effect of Fe_2 DAC to **a,** Newcastle disease virus (NDV), **b,** SARS-CoV-2 and **c,** Vesicular stomatitis virus (VSV)**. d,** Fluorescence images of BHK-21 cells infected by VSV after incubation with different concentrations of Fe_2 DAC. Scale bar = 100 μm. Data are presented as means±SD (n=3 for a and b, while n=5 for c). The significant difference was evaluated by a two-tailed unpaired t-test. *$p < 0.05$, **$p < 0.01$, and ***$p < 0.001$.

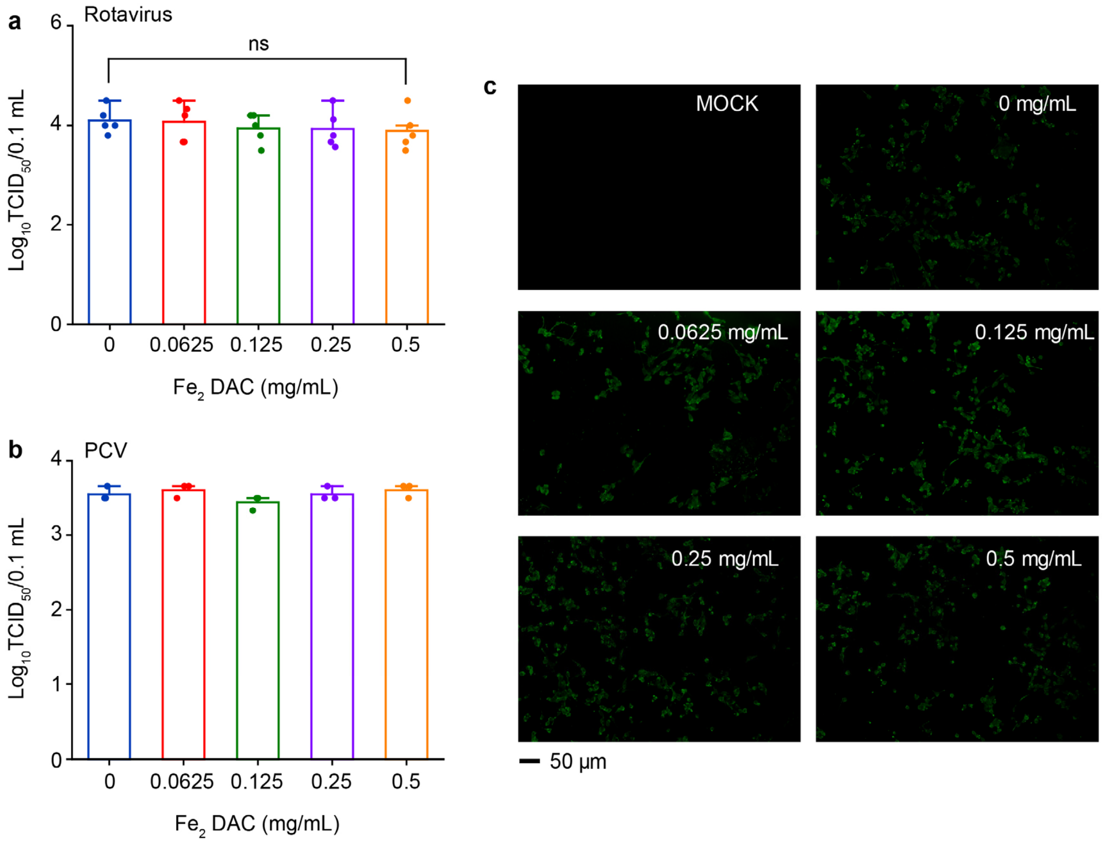

Supplementary Fig. 15. Antiviral effect of Fe_2 DAC to non-enveloped virus. Antiviral effect of Fe_2 DAC to **a,** Rotavirus and **b,** Porcine circovirus (PCV)**. c,** Fluorescence images of PK-15 cells infected by PCV after incubation with different concentrations of Fe_2 DAC. Scale bar = 50 μm. Data are presented as means±SD (n=5 for a and n=3 for b).

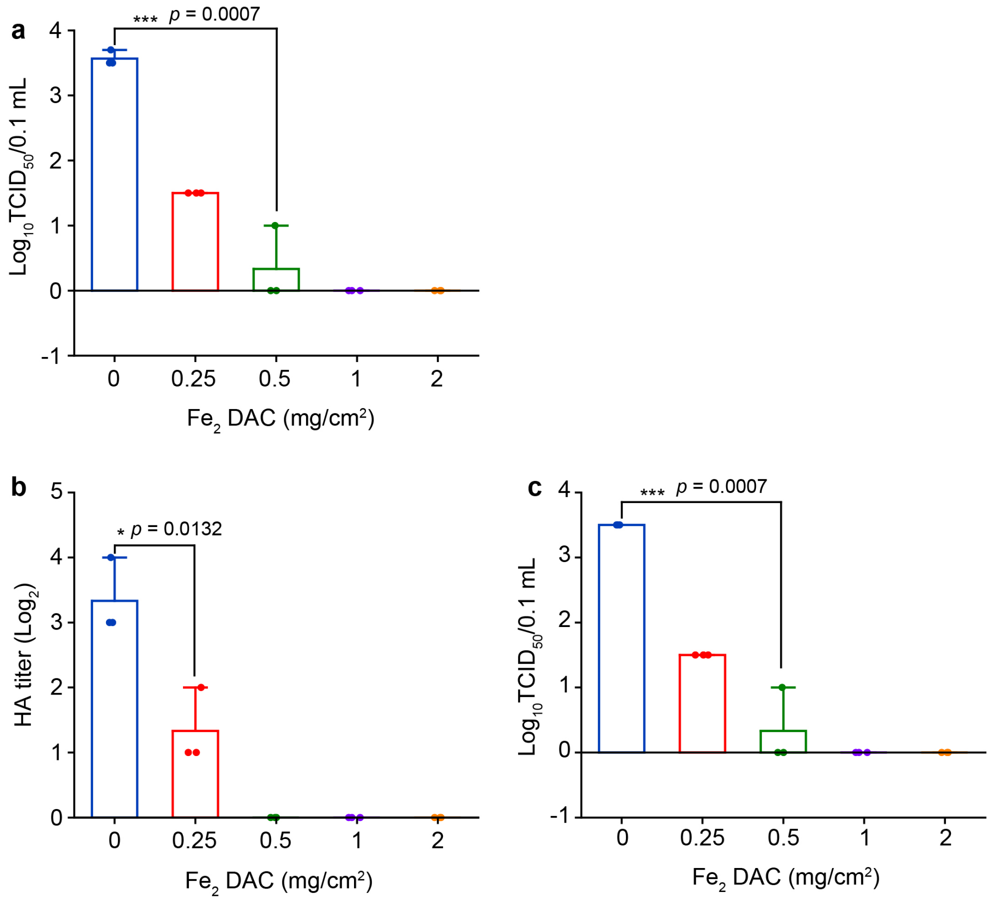

Supplementary Fig. 16. a, $TCID_{50}$ titer of H1N1 IAVs treated with Fe_2 DAC coated on nonwoven (90 min). **b,** HA titer of H1N1 IAVs treated with Fe_2 DAC coated on gauze (90 min). **c,** $TCID_{50}$ titer of H1N1 IAVs treated with Fe_2 DAC coated on gauze (90 min). Data are presented as means±SD (n=3 independent measurements). The significant difference was evaluated by a two-tailed unpaired t-test. $*p < 0.05$ and $***p < 0.001$.

3. Supplementary Tables 1-4

Supplementary Table 1. Crystal data and structure refinements for Fe_2L.

**Fe_2L (CCDC No. 2078111)**			
**Empirical formula**	$Fe_2C_{26}H_{34}N_4O_4Cl_2$	**volume/Å^3**	1366.75(15)
**Formula weight**	649.17	***Z***	2
**Crystal system**	monoclinic	**ρ_{calc}g/cm^3**	1.577
**Space group**	$P2_1/c$	**μ/mm^{-1}**	10.645
***a*/Å**	9.2635(5)	***F*(000)**	672
***b*/Å**	15.8169(6)	**Reflections collected**	6680
***c*/Å**	10.1803(6)	**Independent reflections**	2795
**α/°**	90	**Goodness-of-fit on F^2**	1.036
**β/°**	113.611(7)	**Final R_1^a [$I \geq 2\sigma(I)$]**	0.0325
**γ/°**	90	**Final wR_2^b [$I \geq 2\sigma(I)$]**	0.0719

$^aR_1 = \Sigma||F_0| - |F_c||/\Sigma|F_0|$. $^bwR_2 = |\Sigma w(|F_0|^2 - |F_c|^2)|/\Sigma|w(F_0)^2|^{1/2}$, where $w = 1/[\sigma^2(F_0^2) + (aP)^2 + bP]$. $P = (F_0^2 + 2F_c^2)/3$.

Supplementary Table 2. Fitting results of Fe *K*-edge FT-EXAFS spectrum of Fe_2L and Fe_2 DAC.

Sample	Scattering pair	CN	R (Å)	σ^2 (10^{-3}Å^2)	ΔE	R-factor
Fe_2L	Fe-O	2.1	2.07	2.0	9.1	0.016
	Fe-N	2.2	2.06	1.1	9.1	
	Fe-Fe	1.1	3.14	4.9	9.1	
	Fe-C	2.1	3.01	3.7	9.1	
Fe_2 DAC	Fe-O	2.0	2.10	2.0	9.0	0.009
	Fe-N	2.2	2.08	1.1	9.0	
	Fe-Fe	1.2	3.14	3.9	9.0	
	Fe-C	3.1	3.00	1.0	9.0	

N is coordination number, R is the distance between absorber and backscatter atoms, σ^2 is Debye-Waller factor to account for both thermal and structural disorders, ΔE_0 is inner potential correction; R factor indicates the goodness of the fit. Error bounds (accuracies) that characterize the structural parameters obtained by EXAFS spectroscopy are estimated as N ± 20%, R ± 1%, σ^2 20%, ΔE_0 ± 20%.

Supplementary Table 3. The Michaelis-Menten constant (K_m) and maximum reaction rate (V_{max}) of as-prepared Fe_2 DAC, Fe SAC and HRP for peroxidase-like (POD-like) catalysis.

TMB substrate	[E/Fe] (M)	K_m (M)	V_{max} (M s^{-1})	K_{cat} (s^{-1})	K_{cat}/K_m (M^{-1} s^{-1})
Fe_2 DAC	2.13×10^{-7}	3.61×10^{-7}	5.23×10^{-7}	2.46	6.81×10^{6}
Fe SAC	2.25×10^{-7}	3.64×10^{-7}	8.56×10^{-8}	0.38	1.05×10^{6}
HRP	2.50×10^{-12}	4.69×10^{-7}	3.16×10^{-8}	1.27×10^{4}	2.70×10^{10}

[E/Fe] is the molar concentration of the Fe active sites of the nanozymes or HRP enzyme, which was chosen to obtain the well-fitted Michaelis-Menten plots while varying the substrate concentrations. K_m is the Michaelis constant, V_{max} is the maximal reaction velocity and k_{cat} is the catalytic constant, where $k_{cat} = V_{max}/[E/Fe]$. The k_{cat}/K_m value indicates the catalytic efficiency.

Supplementary Table 4. The Michaelis-Menten constant (K_m) and maximum reaction rate (V_{max}) of as-prepared Fe_2 DAC and Fe SAC with TMB as the substrates for oxidase-like (OXD-like) catalysis.

	**[E/Fe] (M)**	**K_m (M)**	**V_{max} (M s^{-1})**	**K_{cat} (s^{-1})**	**K_{cat}/K_m (M^{-1} s^{-1})**
Fe_2 DAC	2.13×10^{-6}	8.34×10^{-4}	6.15×10^{-7}	2.89×10^{-1}	3.47×10^{2}
Fe SAC	2.25×10^{-6}	8.53×10^{-4}	3.30×10^{-7}	1.46×10^{-1}	1.71×10^{2}

[E/Fe] is the molar concentration of the Fe active sites, K_m is the Michaelis constant, V_{max} is the maximal reaction velocity and k_{cat} is the catalytic constant, where $k_{cat} = V_{max}$/[E/Fe]. The k_{cat}/K_m value indicates the catalytic efficiency of the nanozymes.

4. Supplementary References

1 Ravel, B. & Newville, M. ATHENA, ARTEMIS, HEPHAESTUS: data analysis for X-ray absorption spectroscopy using IFEFFIT. *J. Synchrotron Radiat.* **12**, 537–541 (2005).

2 Sheldrick, G. M. Crystal structure refinement with SHELXL. *Acta Cryst.* **C71**, 3–8 (2015).

3 Dolomanov O., *et al*. OLEX2: a complete structure solution, refinement and analysis program. *J. Appl. Cryst.* **42**, 339–341 (2009).

4 Bagus, P. S. *et al.* Combined multiplet theory and experiment for the Fe 2*p* and 3*p* XPS of FeO and Fe2O3. *J. Chem. Phys.* **154**, 094709 (2021).

5 Oida, S., McFeely, F. R. & Bol, A. A. X-ray photoelectron spectroscopy study on Fe and Co catalysts during the first stages of ethanol chemical vapor deposition for single-walled carbon nanotube growth. *J. Appl. Phys.* **109**, 064304 (2011).

6 Marshall-Roth, T. *et al.* A pyridinic Fe-N(4) macrocycle models the active sites in Fe/N-doped carbon electrocatalysts. *Nat. Commun.* **11**, 5283 (2020).
