# Supplementary material for: Diatomic iron nanozyme with lipoxidase-like activity for efficient inactivation of enveloped virus
Source: Nat Commun. 2023 Nov 11;14:7312. doi: 10.1038/s41467-023-43176-4 (PMC10640610; doi:10.1038/s41467-023-43176-4)
Supplement: Supplementary file 2 — Reporting Summary [file 41467_2023_43176_MOESM2_ESM.pdf]

nature portfolio

Corresponding author(s): Zhiqiang Niu

Last updated by author(s): Oct 20, 2023

Reporting Summary

Nature Portfolio wishes to improve the reproducibility of the work that we publish. This form provides structure for consistency and transparency in reporting. For further information on Nature Portfolio policies, see our Editorial Policies and the Editorial Policy Checklist.

Statistics

For all statistical analyses, confirm that the following items are present in the figure legend, table legend, main text, or Methods section.

n/a	Confirmed	
☐	☒	The exact sample size (n) for each experimental group/condition, given as a discrete number and unit of measurement
☐	☒	A statement on whether measurements were taken from distinct samples or whether the same sample was measured repeatedly
☐	☒	The statistical test(s) used AND whether they are one- or two-sided<br>*Only common tests should be described solely by name; describe more complex techniques in the Methods section.*
☐	☒	A description of all covariates tested
☐	☒	A description of any assumptions or corrections, such as tests of normality and adjustment for multiple comparisons
☐	☒	A full description of the statistical parameters including central tendency (e.g. means) or other basic estimates (e.g. regression coefficient) AND variation (e.g. standard deviation) or associated estimates of uncertainty (e.g. confidence intervals)
☐	☒	For null hypothesis testing, the test statistic (e.g. F, t, r) with confidence intervals, effect sizes, degrees of freedom and P value noted<br>*Give P values as exact values whenever suitable.*
☒	☐	For Bayesian analysis, information on the choice of priors and Markov chain Monte Carlo settings
☒	☐	For hierarchical and complex designs, identification of the appropriate level for tests and full reporting of outcomes
☒	☐	Estimates of effect sizes (e.g. Cohen's d, Pearson's r), indicating how they were calculated

Our web collection on statistics for biologists contains articles on many of the points above.

Software and code

Policy information about availability of computer code

Data collection	Hitachi SU-8000; Hitachi-S4800; Hitachi HT7700; FEI Tecnai Spirit; JEOL JEM-ARM200F; Rigaku RU-200b; Thermo IRIS Intrepid II ICP-OES; TOF.SIMS 5-100 (ION-TOF GmbH); Omicron XPS System using Al Kα X-rays; Beijing Synchrotron Radiation Facility (BSRF); SuperNova charge-coupled device (CCD) X-ray diffractometer;Vario EL III Elemental Analyzer (Elementar, Germany); ESR spectrometer (Bruker A300); Chirascan Plus (Applied Photophysics, UK); Victor NivoTM Multimode Plate Reader (PerkinElmer, USA); Tanon-4600SF; FACSCalibur (BD); UH4150 Spectrophotometer (Direct Light Detector)
Data analysis	FlowJo (v. 10.6, BD Life Sciences); GraphPad Prism (v. 9.3); Image J software; the ATHENA program integrated within the IFEFFIT (1.2.12) software packages; the SHELXL-2018 software package; OLEX2 program; Origin 2021

For manuscripts utilizing custom algorithms or software that are central to the research but not yet described in published literature, software must be made available to editors and reviewers. We strongly encourage code deposition in a community repository (e.g. GitHub). See the Nature Portfolio guidelines for submitting code & software for further information.

Data

Policy information about availability of data

All manuscripts must include a data availability statement. This statement should provide the following information, where applicable:

- Accession codes, unique identifiers, or web links for publicly available datasets
- A description of any restrictions on data availability
- For clinical datasets or third party data, please ensure that the statement adheres to our policy

The data generated in this study are provided in the Supplementary Information/Source Data file (Figshare DOI: 10.6084/m9.figshare.22651555).

Research involving human participants, their data, or biological material

Policy information about studies with human participants or human data. See also policy information about sex, gender (identity/presentation), and sexual orientation and race, ethnicity and racism.

Reporting on sex and gender	n/a
Reporting on race, ethnicity, or other socially relevant groupings	n/a
Population characteristics	n/a
Recruitment	n/a
Ethics oversight	n/a

Note that full information on the approval of the study protocol must also be provided in the manuscript.

Field-specific reporting

Please select the one below that is the best fit for your research. If you are not sure, read the appropriate sections before making your selection.

☒ Life sciences ☐ Behavioural & social sciences ☐ Ecological, evolutionary & environmental sciences

For a reference copy of the document with all sections, see nature.com/documents/nr-reporting-summary-flat.pdf

Life sciences study design

All studies must disclose on these points even when the disclosure is negative.

Sample size	Generally, experiments involving cells were repeated independently on different days using cells of different passage number at least 3 times (biological replicates). For each biological replicate the mean derived from technical replicates (n:3-6) is shown as an individual data point. In vitro chemistry experiments were repeated at least three times.
Data exclusions	No data were excluded.
Replication	Experiments were repeated at least three times independently, and experimental findings were reproducible.
Randomization	Samples were randomly allocated to corresponding experimental groups.
Blinding	The experiments and results analyses were performed by multiple researchers, who had minimal information of sample identification. However, samples were not formally blinded.

Reporting for specific materials, systems and methods

We require information from authors about some types of materials, experimental systems and methods used in many studies. Here, indicate whether each material, system or method listed is relevant to your study. If you are not sure if a list item applies to your research, read the appropriate section before selecting a response.

Materials & experimental systems

n/a	Involved in the study	
	x	Antibodies
	x	Eukaryotic cell lines
x		Palaeontology and archaeology
x		Animals and other organisms
x		Clinical data
x		Dual use research of concern
x		Plants

Methods

n/a	Involved in the study	
x		ChIP-seq
	x	Flow cytometry
x		MRI-based neuroimaging

Antibodies

Antibodies used	The following antibodies were used: anti-Neuraminidase (NA) (11058-MM07, Sino Biological), anti-Hemagglutinin (HA) (11684-MM03, Sino Biological), anti-nucleoprotein (NP) (40205-MM16, Sino Biological). All primary antibodies were used 1:1000 dilution for western blot and 1:300 dilution for flow cytometry. Conjugated antibody: HRP-conjugated goat anti-mouse secondary antibodies (31430, Thermo Fisher), fluorescein Alexa Fluor 488-conjugated goat anti-mouse IgG(A-11001, Invitrogen). HRP-conjugated antibodies were used at 1:1000 dilution for western blot. Fluorescein-conjugated antibody was used at 1:400 dilution for flow cytometry.
Validation	All data can be obtained from the corresponding official websites.

Eukaryotic cell lines

Policy information about cell lines and Sex and Gender in Research

Cell line source(s)	The M90 cell line (NBL-2) and African green monkey kidney cell (Vero) were purchased from ATCC.
Authentication	The cell line was not independently authenticated.
Mycoplasma contamination	All cell lines were tested negative for mycoplasma contamination.
Commonly misidentified lines (See ICLAC register)	No commonly misidentified cell lines were used.

Flow Cytometry

Plots

Confirm that:

- [x] The axis labels state the marker and fluorochrome used (e.g. CD4-FITC).
- [x] The axis scales are clearly visible. Include numbers along axes only for bottom left plot of group (a 'group' is an analysis of identical markers).
- [x] All plots are contour plots with outliers or pseudocolor plots.
- [x] A numerical value for number of cells or percentage (with statistics) is provided.

Methodology

Sample preparation	M90 cell line could be adherently grown in DMEM culture medium containing 10 % fetal calf serum and 1% penicillin and streptomycin. Cells were collected by centrifugation and resuspended in PBS.
Instrument	The data was collected by BD FACSCalibur.
Software	The data was analyzed by FlowJo.
Cell population abundance	We collected 10 000 cells per sample for testing.
Gating strategy	Forward versus side scatter (FSC vs. SSC) was used to gate on live single cells.

- [x] Tick this box to confirm that a figure exemplifying the gating strategy is provided in the Supplementary Information.
